# Molecular Basis of Alcohol-Related Gastric and Colon Cancer

**DOI:** 10.3390/ijms18061116

**Published:** 2017-05-24

**Authors:** Hye-Kyung Na, Ja Young Lee

**Affiliations:** Department of Food Science and Biotechnology, College of Knowledge-Based Services Engineering, Sungshin Women’s University, Seoul 01133, Korea; unun89@naver.com

**Keywords:** alcohol, acetaldehyde, gastric cancer, colon cancer, polymorphism, reactive oxygen species

## Abstract

Many meta-analysis, large cohort studies, and experimental studies suggest that chronic alcohol consumption increases the risk of gastric and colon cancer. Ethanol is metabolized by alcohol dehydrogenases (ADH), catalase or cytochrome P450 2E1 (CYP2E1) to acetaldehyde, which is then further oxidized to acetate by aldehyde dehydrogenase (ALDH). Acetaldehyde has been classified by the International Agency for Research on Cancer (IARC) as a Group 1 carcinogen to humans. The acetaldehyde level in the stomach and colon is locally influenced by gastric colonization by *Helicobacter pylori* or colonic microbes, as well as polymorphisms in the genes encoding tissue alcohol metabolizing enzymes, especially ALDH2. Alcohol stimulates the uptake of carcinogens and their metabolism and also changes the composition of enteric microbes in a way to enhance the aldehyde level. Alcohol also undergoes chemical coupling to membrane phospholipids and disrupts organization of tight junctions, leading to nuclear translocation of β-catenin and ZONAB, which may contributes to regulation of genes involved in proliferation, invasion and metastasis. Alcohol also generates reactive oxygen species (ROS) by suppressing the expression of antioxidant and cytoprotective enzymes and inducing expression of CYP2E1 which contribute to the metabolic activation of chemical carcinogens. Besides exerting genotoxic effects by directly damaging DNA, ROS can activates signaling molecules involved in inflammation, metastasis and angiogenesis. In addition, alcohol consumption induces folate deficiency, which may result in aberrant DNA methylation profiles, thereby influencing cancer-related gene expression.

## 1. Introduction

According to the GLOBOCAN estimates for 2012, there were 14.1 million new cancer cases, 8.2 million cancer deaths and 32.6 million people living with cancer (within five years of diagnosis) [1]. Many epidemiological investigations have shown that the prevalence and mortality of cancer are higher in men than in women. Such disparities can be attributed to gender-specific lifestyle and behavioral characteristics which can influence the effects of exposure to genotoxins.

According to the World Health Organization (WHO) global status report on alcohol and health in 2014, 5.1% of the global burden of diseases is attributable to alcohol consumption [2]. Moreover, alcohol intake is higher in men than women in most countries. Chronic and frequent consumption of alcoholic beverages has been considered to be a risk factor in the etiology of various cancers [3]. Especially, alcohol has been associated with increased risk of gastrointestinal (GI) cancer that occurs in oral cavity, oesophagus, liver, stomach, colon and rectum, etc. [4]. The epidemiological, experimental and clinical studies have revealed that alcohol abuse is a leading cause of cirrhosis which, in turn, is linked with an increased risk of liver cancer [5]. People infected with the hepatitis B virus (HBV) or hepatitis C virus (HCV) have a high risk of developing chronic hepatitis, cirrhosis, and liver cancer [6]. The risk is even higher if they are heavy drinkers (at least six standard drinks a day) [7,8]. Some part of the alcohol which is ingested orally does not enter the systemic circulation but initially undergoes metabolism in the stomach. This first phase metabolism could modulate alcohol toxicity. However, the majority of ethanol is rapidly passed into the duodenum from the stomach, especially in the fasting state [9]. The recent meta-analysis and results of a case-cohort study indicate that alcohol consumption is a risk factor for gastric cancer [10,11,12]. In this context, it is noticeable that in South Korea, gastric cancer was the second most common malignancy and the third most common cause of cancer-associated death in 2014 [13], which is likely to be linked to relatively heavy alcohol consumption in the country.

Colorectal cancer (CRC) is the third most common cancer in the world with nearly 1.4 million new cases diagnosed in 2012. Countries with high incidence rates include South Korea, Slovakia, Hungary, and other parts of Europe [1]. Especially, in Korea, colon cancer incidence and mortality dramatically increased during the last decade with an annual percentage change of 6% (6.5% in males and 5.3% in females) from 1999 to 2011 [13]. Multiple factors, such as high consumption of red meats and insufficient intake of fibers and folate, are associated with the etiology of CRC. Alcohol consumption is also recognized as one of the major risk factors for CRC development [14,15,16]. According to the data from the Korean Multi-Center Cancer Cohort study, longer duration and higher average amount of alcohol consumption account for elevated risk of CRC in men (HR 1.93 [1.17–3.18] for ≥30 years of consumption compared to non-drinkers; HR 2.24 [1.31–3.84] for ≥30 g/day) [17]. World Cancer Research Fund and American Institute for Cancer Research have reported that consumption of more than 30 g/day of ethanol can be a cause of CRC [18]. In particular, among Korean women, 0.5% (464 cancer cases) of incident cancers and 0.1% (32 deaths) of cancer deaths were attributable to alcohol consumption [19]. The sex- and cancer-specific population attributable fractions for CRC incidence were the highest (4.2%) and that for breast cancer incidence was only 0.2% [19].

## 2. Alcohol Metabolism

**Figure 1:**
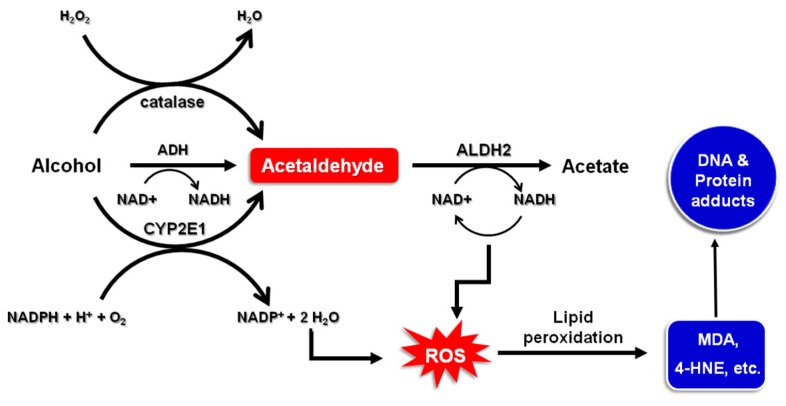
Oxidative pathways of alcohol metabolism. ADH, CYP2E1, and catalase all contribute to oxidative metabolism of alcohol to generate the acetaldehyde. ADH, present in the cytosol, converts alcohol to acetaldehyde, which is coupled with reduction of nicotinamide adenine dinucleotide (NAD^+^) to NADH. NADH is reoxidized to NAD^+^ with concomitant generation of ROS. CYP2E1 metabolizes ethanol to acetaldehyde at elevated ethanol concentrations. Acetaldehyde is metabolized mainly by ALDH2 to form acetate and NADH. Accumulation of ROS resulting from the alcohol oxidation leads to formation of lipid peroxides which, in turn, cause modification of proteins and DNA.

Many factors influence ethanol absorption, disposition and metabolism. These include gender, age, ethnicity and body weight. Ethanol is metabolized by alcohol dehydrogenases (ADH), catalase or cytochrome P450 2E1 (CYP2E1) to acetaldehyde which is then further oxidized to acetate by aldehyde dehydrogenase (ALDH) (Figure 1). Among all classes of ADH and ALDH isozymes, ADH1B, ADH1C and ALDH2 are mainly involved in ethanol metabolism [20,21]. Acetaldehyde has been classified by the International Agency for Research on Cancer (IARC) as a Group 1 carcinogen to humans [22]. Acetaldehyde has been known to induce DNA damage in the digestive tract [23]. Polymorphisms in ethanol- and acetaldehyde-metabolizing enzymes, especially ADH and ALDH, have been closely associated with ethnic and individual differences in susceptibility to alcohol-related cancers.

In East Asian populations, there is a variant of ALDH2, a major acetaldehyde eliminating enzyme, resulting from the replacement of glutamate (Glu) at the position 487 with lysine (Lys) [24,25]. The Glu allele (ALDH2*1) encodes a protein with normal catalytic activity, whereas the Lys allele (ALDH2*2) encodes a catalytically inactive enzyme. As a result, Lys/Lys homozygotes have no detectable ALDH2 activity. In addition, a mutant form of ADH (ADH1B*2) is highly prevalent among Eastern Asians [25]. In line with this notion, the levels of acetaldehyde-derived DNA adducts were significantly elevated in the blood of alcoholic patients with the ALDH2-deficient genotype compared to those with the ALDH2 wild type genotype when they consumed equivalent amounts of alcohol [26].

Initial ethanol metabolism by CYP2E1 and the re-oxidation of NADH via the electron transport chain in the mitochondria give rise to the formation of reactive oxygen species (ROS) [27]. Ethanol metabolism can drastically change the NADH/NAD^+^ ratio which functions as a metabolic determinant that controls gene activation or silencing [27]. Oxidative stress caused by ROS formed during ethanol oxidation has been known to be a pathogenic event implicated in pathogenesis of majority of alcohol-induced toxicity [28]. Ethanol treatment induces expression of CYP2E1 involved in metabolic activation of some gastric carcinogens, including nitrosamines. It also suppresses the expression of antioxidant enzymes and other cytoprotective proteins including superoxide dismutase 1 and peroxiredoxin, thereby accelerating the generation of ROS [29]. Accumulation of ROS and electrophilic species, such as acetaldehyde and lipid peroxidation products (e.g., 4-hydroxynonenal; 4-HNE) formed during ethanol metabolism, can cause DNA damage, thereby initiating malignant transformation [30]. Furthermore, there is a vicious cycle between ROS-induced oxidative stress and inflammation which is responsible for promotion of carcinogenesis. Thus, oxidative stress has been known to stimulate the proliferation of initiated cells through activation of inflammatory signaling and vice versa. In addition, alcohol can augment genotoxic effects of carcinogens by decreasing their elimination and/or stimulating metabolic activation [31]. Alcohol has also been shown to affect one-carbon metabolism via suppression of folate utility and consequently hypermethylation of CpG islands of tumor suppressor genes [32].

According to the WHO global status report on alcohol and health in 2014, the amount of pure alcohol consumption by South Korean is 12.3 L per capita per year, which is the largest in the world [2]. Although the association between consumption of alcohol and the risk of GI cancer is well known, the molecular basis of effects of alcohol on stomach and colon cancers remain poorly understood. This review highlights effects of alcohol on development and pathogenesis of gastric and CRC and their underlying molecular mechanisms.

## 3. Genetic Polymorphism of Alcohol Metabolizing Enzymes

### 3.1. Polymorphism Associated with Gastric Cancer

The several isoenzymes of ADH have been found in the stomach, and gastric ADH activity has been reported to be affected by several factors, including ethnicity, age, gender and certain drugs (e.g., cimetidine and ranitidine) [33]. According to the Japan Public Health Center-based prospective study, there is no association of alcohol consumption and polymorphisms of ADH1B (rs1229984), ADH1C (rs698) and ALDH2 (rs671) with gastric cancer risk [34]. In addition, ADH2 and ALDH2 polymorphism and alcohol drinking do not appear to be linked each other for the development of stomach cancer in Chinese males [35]. However, ADH1C G allele carriers who drink ≥150 g/week of ethanol had a 2.5-fold increased risk of gastric cancer development (OR = 2.54, 95% CI = 1.05−6.17) relative to AA genotype carriers who do not drink at all or drink <150 g/week (P for interaction = 0.02) [34]. The recent meta-analysis suggests that *ALDH2* and *ADH1* genetic polymorphisms may play crucial roles in the pathogenesis of gastric cancer. However, *ADH2* genetic polymorphisms may not be an important determinant of susceptibility to gastric cancer [36].

Drinking alcohol caused DNA damage in mouse stomach, which was ALDH2 genotype-dependent [37]. Thus, the level of DNA adducts formed followed in the order of ALDH2−/−, ALDH2+/−, and ALDH2+/+ mice treated with ethanol. Notably, ALDH2 A allele carriers who drink ≥150 g/week have an increased risk of gastric cancer (OR = 2.08, 95% CI = 1.05–4.12) relative to GG genotype carriers who drink 0 to <150 g/week (*p* for interaction = 0.08) [34]. This result suggests that ALDH2 deficiency may accelerate gastric carcinogenesis.

### 3.2. Polymorphism Associated with Colon Cancer

Alcohol ingested orally is transported to the colon by blood circulation after absorption, and intracolonic ethanol levels are equal to those in the blood. Rat colonic mucosa was found to possess detectable ALDH activity, but the activity was generally low compared with that in liver, stomach, and small intestine [38]. In addition, cytosolic ADH activity in colonic mucosa was found to be approximately 6 times lower than in the liver and about one-half of gastric ADH activity [38]. As in the case of gastric cancer, polymorphisms or mutations of genes encoding enzymes responsible for acetaldehyde generation or elimination are associated with ethnic or individual differences in the susceptibility to alcohol-associated CRC. The frequencies of the mutant ALDH2*2 allele were significantly higher in Japanese alcoholics with colon cancer (21.7%) than in those cancer-free alcoholics (9.0%) [39]. However, according to the large population-based case-control study conducted in Israel, there is no association between *ALDH2* rs886205 polymorphism and CRC risk [40]. A number of studies have explored the association between the ALDH2*2 polymorphism and risk of colorectal cancer; however, the results are inconsistent. In addition, polymorphism in *ADH1B* rs1229984 has been found to be directly associated with CRC risk and it also shows an indirect effect, mediated through alcohol consumption [40]. A matched case-control study conducted in Japan showed that the ADH2 Arg allele was found to be associated with an increased CRC risk. In contrast, no apparent links were observed with the ALDH2 genotypes [41].

## 4. Mechanisms Underlying Alcohol-Induced Gastric and Colonic Carcinogenesis

**Figure 2:**
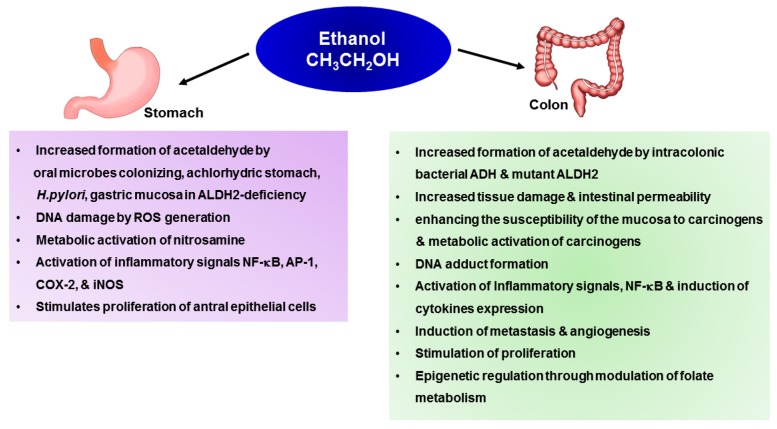
The molecular mechanisms underlying alcohol-induced stomach and colon carcinogenesis.

Several mechanisms have been suggested to delineate alcohol consumption and cancer development. It is speculated that both genetic and epigenetic mechanisms are involved in alcohol-induced carcinogenesis. An understanding of the mechanisms by which ethanol induces neoplastic transformation is important for development of appropriate strategies in the management of alcohol-associated cancers. Chronic ethanol consumption may initiate or promote carcinogenesis via multiple mechanisms [42]. Ethanol itself is not carcinogenic, but available data suggest that acetaldehyde and ROS have genotoxic or tumor promoting effects. Thus, co-administration of ethanol and cyanamide, a potent acetaldehyde dehydrogenase inhibitor, increased the incidences of tumors in acetoxymethylmethylnutrisamine treated rats, lending further support to the notion that acetaldehyde could be involved in the ethanol-associated carcinogenesis [43]. Other carcinogenetic mechanisms include nutritional deficits, changes in DNA methylation, and impaired immune surveillance [44]. Figure 2 summarizes the molecular mechanisms underlying alcohol-induced stomach and colon carcinogenesis (Figure 2).

### 4.1. Gastric Cancer

#### 4.1.1. Generation of Acetaldehyde by Microbiome

The intragastric acetaldehyde level is locally regulated not only by gastric mucosal ADH and ALDH2, but also by microbes colonizing stomach and saliva [45]. *Helicobacter pylori* (*H. pylori*) infection is known to cause gastritis which is implicated in the etiology of gastric cancer. Individuals infected with *H. pylori* have a 1% to 2% risk of acquiring stomach cancer [46]. It is interesting to note that some *H. pylori* strains retain substantial cytosolic ADH activity and produce considerable amounts of acetaldehyde when incubated with ethanol [47]. Ten ALDH2-active and the same number of ALDH2-deficient *H. pylori*-negative healthy volunteers were subjected to alcohol infusion via the nasogastric tube into the stomach [45]. There was a dramatic increase in the levels of acetaldehyde in the gastric juices of ALDH2-deficient subjects, compared with the ALDH2-active individuals [45].

Besides its formation by *H. pylori* in the gastric mucosa, acetaldehyde can also be produced from ethanol by oral bacteria, and high concentrations of aldehyde have been observed in human saliva after ethanol consumption [23]. In addition, chronic smoking modifies oral flora to produce more acetaldehyde from ingested alcohol [48]. Moreover, acetaldehyde is also present in the main stream tobacco smoke [49]. It has been reported that smokers have 1.62-fold higher risk of gastric cancer (95% CI: 1.50–1.74) than nonsmokers in Chinese population [50]. Notably, cigarette smoking and drinking alcohol have been considered to have synergistic effects on the development of stomach cancer [50].

#### 4.1.2. Inflammation Induced by Alcohol

Inflammation has been known to accelerate the tumor promotion and progression. Intragastric administration of ethanol to male Sprague-Dawley rats caused significant damage in gastric mucosa, which was accompanied by upregulation/activation of some enzymes and transcription factors involved in proinflammatory signaling [51]. These include elevated expression of cyclooxygenase-2 (COX-2) and inducible nitric oxide synthase (iNOS) as well as transient activation of the redox-sensitive transcription factors, NF-κB and AP-1 and also mitogen-activated protein kinases (MAPKs) [51]. Atrophic gastritis leads to microbial colonization of the stomach, which could enhance microbial production of acetaldehyde from ethanol [52]. A multivariate analysis showed that *H. pylori*-induced chronic atrophic gastritis (CAG) and the ALDH2*1/2*2 genotype were independently associated with the risk of developing gastric carcinoma in alcoholic Japanese men [53]. Thus, combination of *H. pylori*-derived CAG and ALDH2*1/2*2 exacerbated the progression to gastric carcinoma (OR = 39.2 for severe CAG plus ALDH2*1/2*2) [53].

Colonization of *H. pylori* in the stomach is often accompanied by achlorhydric atrophic gastritis which is a well-known risk factor for gastric cancer. Achlorhydric atrophic gastritis is characterized by impaired gastric secretion of essential digestive substances, such as hydrochloric acid and pepsin. Ethanol metabolism by microbiome in the stomach leads to high intragastric acetaldehyde production in patients with achlorhydric atrophic gastritis [52]. In another study, incubation of gastric juice samples from atrophic gastritis patients with 1% ethanol resulted in a 7.6-fold increase in the acetaldehyde production, compared with the control samples [52].

#### 4.1.3. Metabolic Activation of Carcinogens and Their DNA Adduct Formation

Nitrosamins are potent carcinogens which are widely present in the foods and environments. Nitrosamins are also formed in the stomach through interaction between nitrite and secondary amines derived from proteins. Ethanol suppresses the hepatic clearance of nitrosamines. *N*-nitrosodimethylamine (NMDA) produced from sodium nitrite and dimethylamine in the stomach is suspected as a human gastric cancer initiator [54]. Adult monkeys given 0.1 mg/kg NMDA by gavage showed a high level of the genotoxic DNA lesion *O*^6^-methylguanine (*O*^6^-MeG) in stomach [54]. Of note, alcohol further enhanced the NMDA-induced DNA adduct formation in the stomach of the monkeys [54]. Ethanol also promotes gastric carcinogenesis by stimulating proliferation of the antral epithelial cells [55]. Thus, prolonged administration of ethanol resulted in a significant increase in the incidence and the number of gastric cancers of the glandular stomach in Wistar rats after *N*-methyl-*N*′-nitro-*N*-nitrosoguanidine treatment [55].

### 4.2. Colon Cancer

#### 4.2.1. Generation of Acetaldehyde by Microbiome

Besides metabolism by ADH activity in colonic mucosa, ethanol can also undergo oxidative metabolism by intracolonic bacterial ADH to yield acetaldehyde. Incubation of human colonic contents containing microbiome with various concentrations of ethanol at 37 °C resulted in significant accumulation of acetaldehyde [56]. Due to the low ALDH activity of colonic mucosa, substantial amounts of acetaldehyde accumulate in the colon, and this would contribute to the increased prevalence of colonic polyps and CRC which have been found to be linked to heavy alcohol consumption [57]. There was a statistically significant correlation between microbial ADH activity and acetaldehyde production from ethanol in the colon [58]. More than 500 bacterial strains isolated from the feces of Japanese alcoholics were phylogenetically characterized, and their ability to produce acetaldehyde from ethanol beyond the minimum mutagenic concentration was examined [59]. Among these, some obligate anaerobes were found to be potential acetaldehyde accumulators [59]. Ethanol oxidation by intestinal obligate anaerobes under aerobic conditions in the colon and rectum is hence likely to play an important role in the pathogenesis of alcohol-associated colorectal cancer [59]. Alcohol administration gave rise to very high intracolonic acetaldehyde levels in rat, which were markedly decreased by concomitant treatment with ciprofloxacin, an antibiotics [60]. Furthermore, germ-free rats had significantly lower acetaldehyde accumulation in the rectum and in the cecum than conventional animals, and this was paralleled by the number of fecal bacteria in the intestinal segments [43]. In addition, individual variations in human colonic microflora may influence the relative risk of alcohol-related colorectal cancer [61].

#### 4.2.2. Increased Uptake of Carcinogens

A major function of epithelial cells lining the digestive tube and the tight junctions is to provide a barrier against the hostile environment in the gastrointestinal lumen. Dysregulation of the interactions between the gut epithelium and intestinal bacteria leads to the loss of host immune tolerance, and thereby promotes the development of colon cancer. Excessive intake of ethanol changes the composition of enteric microflora, induces the overgrowth of gram-negative bacteria, and disrupts the intestinal epithelial barrier [62]. These result in increased intestinal permeability, and increased accumulation of proinflammatory cytokines, such as tumor necrosis factor (TNF)-α and interleukin (IL)-6 [62]. Ethanol does facilitate the uptake of environmental carcinogens by changing permeability and molecular composition of the GI tract [31]. Alcohol also acts as a solvent that enhances the penetration of carcinogenic compounds into the mucosa.

#### 4.2.3. Metabolic Activation of Carcinogens and Their DNA Adduct Formation

CYP2E1 induced by chronic alcohol consumption plays an important role in alcohol-mediated carcinogenesis [63]. Chronic ethanol administration increased expression of CYP2E1 in the colon of adult male Sprague-Dawley rats [64]. CYP2E1 induction, in turn, stimulates ethanol metabolism, resulting in the generation of ROS and reactive nitrogen species, and has been associated with diminished cellular antioxidant defence capacity [65]. ROS can cause oxidative degradation of membrane lipids. The resulting lipid peroxidation products, such as 4-HNE, can bind to DNA, forming highly carcinogenic exocyclic etheno DNA-adducts in colonic mucosa [30].

CYP2E1 also metabolizes various procarcinogens present in diets and in tobacco smoke to ultimate carcinogenic forms [63]. Chronic ethanol feeding significantly increased the number of aberrant crypt foci (ACF) in colons of rats treated with 1,1-dimethylhydrazine, an organotropic colon carcinogen metabolically activated to form DNA-reactive species [66]. 1,1-Dimethylhydrazine undergoes *N*-oxidation to form azoxymethane (AOM), which is hydroxylated primarily by CYP2E1 to yield methylazoxymethanol (MAM) [67]. MAM is unstable, and subsequently decomposes to yield formaldehyde and a highly reactive methyldiazonium ion, capable of alkylated DNA adducts [67]. Of these, *O*^6^-MeG is the most mutagenic and contributes to colon tumorigenesis [68]. In line with this notion, the colonic levels of *O*^6^-MeG and formation of aberrant crypt foci were significantly lower in male CYP2E1 null mice treated with AOM than in AOM-treated WT mice [67]. Therefore, ethanol-induced CYP2E1 induction contributes to the colon carcinogenesis induced by some chemical carcinogens that undergo metabolic activation by this enzyme [67]. Several experimental studies have also revealed that alcohol can promote colon carcinogenesis. Thus, ethanol treatment significantly enhanced MAM acetate-initiated large bowel carcinogenesis in ACI/N rats [69]. Oral administration of ethanol dramatically increased the rectosigmoidal colon tumor incidence and tumor growth in rat treated with MAM acetate [69].

#### 4.2.4. Inflammation-Associated Tumor Promotion

Sustained inflammatory insults and oxidative stress comprise a vicious circle, which can damage healthy neighboring epithelial and stromal cells over a long period of time, stimulating carcinogenesis [70]. Colon cancer is one of the most well defined malignancies in which inflammation plays a role in multi-stage carcinogenesis. Chronic ethanol feeding promotes AOMplus dextran sulfate sodium (DSS)-induced colonic tumorigenesis potentially by enhancing inflammation in the colonic mucosa of mice [71]. Ethanol causes sustained inflammatory reaction in the colonic mucosa and submucosa as evidenced by infiltration of mono- and some polymorphonuclear cells, leading to formation of lymphoid aggregates, disruption of mucosal integrity and occurrence of some erosive spots on the epithelial surface [71]. Myeloperoxidase and granulocyte receptor-1 are neutrophil markers. Cells expressing these markers were detected in the colonic mucosa of ethanol fed AOM-DSS mice, but not in AOM-DSS mice [71]. In addition, ethanol administration significantly increased the expression of proinflammatory cytokines (IL-1α, IL-6 and TNF-α) and chemokines (CCL5/RANTES, CXCL9/MIG and CXCL10/IP-10) in the colonic mucosa in a precancerous stage. Chronic ethanol feeding significantly increased the number and the size of polyps in the colon of AOM plus DSS treated mice, possibly by augmenting inflammation [71]. In addition to alterations in gut permeability, ethanol elicits a synergistic effect with gut microbiome on production of proinflammatory cytokines, such as TNF and IL-6 [62].

NF-κB plays a critical role in the development of inflammation-associated colon cancer. Following ethanol administration to rats, the amount of its inhibitor, IκB in the cytoplasm decreased in colon, but the localization of p65, the functionally active subunit of NF-κB in the nucleus, increased [72]. As a consequence, the expression of iNOS and content of its product nitric oxide were elevated, leading to inhibition of colonic contraction in rats [72]. Ethanol treatment enhanced production of both interferon γ and IL-4 in the cells from cecal lymph node [73]. In addition, ethanol enhanced arsenic-induced COX-2 expression through generation of ROS and activation of NF-κB [74].

#### 4.2.5. Invasion and Metastasis

Alcohol increased the migration/invasion of colorectal cancer cells (DLD1, HCT116, HT29, and SW480) in a concentration-dependent manner [75]. Monocyte chemoattractant protein-1 (MCP-1), known as chemokine ligand 2 (CCL2), is one of the critical chemokines implicated in the aggressiveness of CRC and could predict poor prognosis [76]. Alcohol increased the expression of MCP-1 and its receptor CCR2 at both protein and mRNA levels in the CRCs, which was associated with enhanced migration of colon cancer cells [75]. An antagonist of CCR2 blocked alcohol-stimulated migration [75]. β-Catenin is involved in regulation of MCP-1 expression [77]. Ethanol caused an initial cytosolic accumulation of β-catenin and its subsequent nuclear translocation by inhibiting glycogen synthase kinase (GSK)3β activity in colon cancer cells [75]. Furthermore, knock-down of MCP-1/CCR2 or β-catenin was sufficient to inhibit alcohol-induced cell migration/invasion. Together, these results suggested that alcohol enhanced migration/invasiveness of CRC by modulating the GSK3β/β-catenin/MCP-1 pathway [75].

Epithelial-mesenchymal transition (EMT) represents one of critical cellular events involved in cancer progression. Alcohol promotes expression of matrix metalloproteinases (MMP-2, -7, -9), and vimentin, and also phosphorylation and nuclear translocation of Snail involved in EMT by increasing epidermal growth factor receptor transactivation in colon cancer cells [78]. In addition, Snail mRNA expression was significantly higher in colonic biopsies from chronic heavy alcohol drinkers as compared to controls [78]. Knockdown of Snail suppressed the expression of vimentin induced by ethanol in colon cancer cells [78]. Acetaldehyde activates Snail in an intestinal epithelium [78]. Activated Snail, in turn, mediates acetaldehyde-induced tight junction disruption and increase in paracellular permeability [79].

#### 4.2.6. Induction of Angiogenesis

Alcohol is involved in angiogenesis of colon cancer. Ethanol dose-dependently increased tube formation of human umbilical vein endothelial cells (HUVECs) on matrigel [80]. Ethanol also stimulated the migration of HUVECs. Moderate consumption of alcohol enhances endothelial angiogenic activity in HUVECs by stimulating a Notch/CBF-1/RBP-JK-Ang1/Tie2-dependent pathway [80].

Ethanol markedly enhanced arsenic-induced tumor angiogenesis which was attributed to intracellular ROS generation, NADPH oxidase activation, and activation of PI3K/Akt and hypoxia-inducible factor 1 alpha (HIF-1α) [81]. Antioxidant enzymes and the HIF-1 inhibitor attenuated arsenic/ethanol-induced tumor angiogenesis [81]. Ethanol feeding elevated the total number of tumors nearly 4-fold and induced expression of P-Smad, vascular endothelial cell growth factor and HIF-1α in the colon of mice treated with AOM and dextran sulfate sodium [71].

Mast cells promote innate immunity to protect against microbial infections and also fine tune angiogenic switching, thereby maintaining vascular network and gut epithelial barrier permeability [82]. Chronic alcohol feeding increased the proportion of mast cells and their activity at the site of polyps and invading borders in the large intestines of *APC*^∆468^ mice [83]. This was accompanied by the increased number and the size of polyps.

#### 4.2.7. Stimulation of Cancer Cell Proliferation

Colorectal cell turn-over is affected by multiple factors including alcohol consumption, diets, smoking or age and is also significantly changed in certain mucosal diseases including CRC [84]. Mucosal hyperregeneration is speculated to increase the risk of CRC by enhancing the susceptibility of the mucosa to carcinogenic insults. Chronic alcohol consumption leads to mucosal cellular hyperregeneration [84]. The proliferating cell nuclear antigen index was increased significantly in the colon after ethanol administration [85]. Alcohol induces rectal hyperregeneration, which was accompanied by an increase in the crypt proliferative compartment and mucosal ornithine decarboxylase activity [43]. Hyperregeneration in gastrointestinal mucosa may hence be a plausible mechanism by which alcohol exerts its tumor promoting or cocarcinogenic effects [86].

Mutation of the *APC* tumor suppressor gene is found in 80% to 90% of sporadic colorectal tumors and in all cases of the inherited form of colon cancer, familial adenomotous polyposis. Ethanol ingestion increased intestinal tumorigenesis in the multiple intestinal neoplasia (*Min*) mouse model of intestinal tumorigenesis [87]. The large majority of β-catenin is recruited at adherens junctions through its interaction with E-cadherin. Therefore, mutations in *APC* or β-*catenin* or any significant disruption of their interaction can result in the nuclear translocation of β-catenin, thereby inducing the expression of numerous genes involved in proliferation events. Ethanol supplementation increased the number of tumors, especially in the distal small bowel of the *Min* mice harboring mutated *APC* gene [87]. Ethanol has also been shown to activate the Wnt/β-catenin signaling in cultured colon cancer cells [75]. Therefore, chronic alcohol consumption augments cell signaling involved in proliferation of colonic mucosa through overactivation of the Wnt/β-catenin axis.

Ethanol could also undergo chemical coupling to membrane phospholipids via the action of phospholipase D, resulting in the conversion of phosphatidylcholine into phosphatidylethanol [88]. Thus, the accumulation of phosphatidylethanol has been detected following chronic ethanol exposure and plays a role in the ethanol-induced alterations of tight junction (TJ) organization [88]. Ethanol caused a progressive disruption of TJ protein zonula occludens-1 (ZO-1) and its displacement from the cellular borders [89]. This resulted in formation of large paracellular openings between the adjacent cells by activating myosin light chain kinase [89]. The Y-Box transcription factor, ZONAB was partly colocalized with ZO-1 at the TJ cytoplasmic plaque. Ethanol disrupted association between ZO-1 and the ZONAB, which led to nuclear translocation of ZONAB in intestinal cells [89]. Incorporation of membrane phosphatidylethanol and disruption of ZO-1 and ZONAB colocalization correlate with increased cell proliferation in the colonic epithelium of ethanol-fed mice and in the adenomas of chronic alcohol consumers [89].

#### 4.2.8. Epigenetic Mechanism

DNA methylation is an important epigenetic determinant of gene expression, and differential methylation has been associated with multiple diseases including cancer. CRC is characterized by both genetic and epigenetic changes (e.g., regional DNA hypermethylation and global DNA hypomethylation). Folate plays a pivotal role in the one-carbon metabolism pathway by providing a methyl group required in a wide range of biochemical reactions, including methylation of DNA [90]. Low dietary intake of folate is a well-known independent risk factor for CRC and is associated with aberrant DNA methylation [91]. The promoter hypermethylation of tumor suppressor genes, such as *APC*, *p14ARF*, *p16INK4A*, *r hMLH1*, *O*^6^-*MGMT*, and *RASSF1A*, was higher in CRCs derived from patients with low folate/high alcohol intake when compared with CRCs from patients with high folate/low alcohol intake [32]. The alcohol dehydrogenase iron containing 1 (ADHFE1) is responsible for the oxidation of 4-hydroxybutyrate to succinate semialdehyde [92]. ADHFE1 was found to be hypermethylated in CRC tissues and colon cancer cells [93]. In addition, mRNA expression levels of ADHFE1 in CRC tissues, compared to adjacent normal tissues, were significantly reduced in elderly drinkers [93]. Ethanol treatment promotes the hypermethylation of ADHFE1 and methylation-mediated silencing of ADHFE1, which is responsible for enhanced cell proliferation of colon cancer cells [93]. It was reported that alcohol administration decreased significantly colonic mucosal folate levels by 48% in rats [60]. Acetaldehyde degrades folate in vitro. Thus, the high levels of acetaldehyde produced from ethanol by gut microbiome are likely to stimulate the break down of folate in the colon, thereby mimicking the effects of folate deficiency on colon carcinogenesis [60]. The serum folate concentrations were significantly different between two ALDH2 genotypes [94]. The reduction of serum folate as a consequence of alcohol drinking was more pronounced in men with ALDH2*1/2*2 than those with ALDH2*1/2*1 [94].

Methylenetetrahydrofolate reductase (MTHFR) is a key folate metabolizing enzyme. Chronic heavy drinking reduces folate levels and inhibits methionine synthase, resulting in the reduction of methionine and *S*-adenosylmethionine and the concurrent increase in homocysteine and *S*-adenosylhomocysteine. *S*-adenosylhomocysteine further inhibits DNA methyltransferases, ultimately resulting in global hypomethylation of DNA [27]. The most common form of genetic hyperhomocysteinemia results from a 677C>T polymorphism (NM_005957.4:c.665C>T, rs1801133) in MTHFR. MTHFR 677TT homozygotes are relatively resistant to development of CRC when folate is high and alcohol is low, but are at a high risk when folate intake is low, or when excess alcohol is consumed [95].

## 5. Conclusions

Population-based and experimental studies suggest that chronic alcohol consumption increases the risk of stomach and colon cancer. Ethanol induces disassembly and displacement of proteins from the cellular borders or perijunctional area. This causes translocation of membrane or junction proteins into cytosol or nucleus and regulates or indirectly influences expression of genes involved in cell proliferation. Intake of ethanol not only causes mucosal damage but also changes the composition of enteric microflora, which disrupts the intestinal epithelial barrier.

Acetaldehyde formed during oxidative ethanol metabolism is suspected to be a human carcinogen. Therefore, deficiency or functional inactivation of enzymes involved in ethanol metabolism, especially ADH and ALDH, is expected to influence the alcohol-associated carcinogenesis. Generation of ROS during ethanol metabolism also activates signaling molecules involved in inflammation, angiogenesis, and migration/metastasis as well as causes DNA damage. Chronic and frequent alcohol consumption also results in the folate deficiency, which may provoke aberrant DNA methylation profiles, thereby influencing cancer-related gene expression.

Overall, genetic polymorphisms of alcohol metabolizing enzymes, tobacco smoking, folate deficiency, and GI microbiome can influence the alcohol-associated stomach and colon carcinogenesis. Therefore, quitting alcohol drinking and tobacco smoking, intaking adequate levels of folate and maintenance of optimal microflora comprise effective strategies to prevent stomach and colon cancer. According to the report of World Cancer Research Found International, it is best not to drink alcohol to prevent a substantial proportion of cancer. If alcoholic drinks are to be consumed, it is recommended to limit consumption to no more than two drinks a day for men and one drink a day for women (one drink contains about 10–15 g of ethanol).

## Abbreviations

GI	Gastrointestinal
CYP2E1	Cytochrome P4502E1
CRC	Colorectal cancer
ADH	Alcohol dehydrogenases
ALDH	Aldehyde dehydrogenase
ROS	Reactive oxygen species
4-HNE	Hydroxynonenal
*H. pylori*	*Helicobacter pylori*
*O*^6^-MeG	*O*^6^-methylguanine
iNOS	Inducible nitric oxide synthase
MAM	Methylazoxymethanol
TNF	Tumor necrosis factor
IL	Interleukin
AOM	Azoxymethane
DSS	Dextran sulfate sodium
MCP-1	Monocyte chemoattractant protein-1
HUVECs	Human umbilical vein endothelial cells
HIF-1α	Hypoxia-inducible factor 1 alpha
TJ	Tight junction
ZO-1	Zonula occludens-1
MTHFR	Methylenetetrahydrofolate reductase
